# Feasibility of improving vascular imaging in the presence of metallic stents using spectral photon counting CT and K-edge imaging

**DOI:** 10.1038/s41598-019-56427-6

**Published:** 2019-12-27

**Authors:** Monica Sigovan, Salim Si-Mohamed, Daniel Bar-Ness, Julia Mitchell, Jean-Baptiste Langlois, Philippe Coulon, Ewald Roessl, Ira Blevis, Michal Rokni, Gilles Rioufol, Philippe Douek, Loic Boussel

**Affiliations:** 1 Hospices Civils de Lyon, Louis Pradel Cardiology Hospital, Lyon, France; 2 Univ Lyon, INSA‐Lyon, Université Claude Bernard Lyon 1, UJM-Saint Etienne, CNRS, Inserm, CREATIS UMR 5220, U1206 Lyon, France; 3 Cardiac surgery, Hospices Civils de Lyon, Lyon, France; 4 CERMEP Centre d’imagerie du vivant, Lyon, France; 5 CT Clinical Science, Philips, Suresnes France; 6 Philips Research Laboratories, Hamburg, Germany; 7 Global Advanced Technologies, CT, Philips, Haifa Israel

**Keywords:** Restenosis, Preclinical research

## Abstract

Correct visualization of the vascular lumen is impaired in standard computed tomography (CT) because of blooming artifacts, increase of apparent size, induced by metallic stents and vascular calcifications. Recently, due to the introduction of photon-counting detectors in the X-ray imaging field, a new prototype spectral photon-counting CT (SPCCT) based on a modified clinical CT system has been tested in a feasibility study for improving vascular lumen delineation and visualization of coronary stent architecture. Coronary stents of different metal composition were deployed inside plastic tubes containing hydroxyapatite spheres to simulate vascular calcifications and in the abdominal aorta of one New Zealand White (NZW) rabbit. Imaging was performed with an SPCCT prototype, a dual-energy CT system, and a conventional 64-channel CT system (B64). We found the apparent widths of the stents significantly smaller on SPCCT than on the other two systems *in vitro* (p < 0.01), thus closer to the true size. Consequently, the intra-stent lumen was significantly larger on SPCCT (p < 0.01). In conclusion, owing to the increased spatial resolution of SPCCT, improved lumen visualization and delineation of stent metallic mesh is possible compared to dual-energy and conventional CT.

## Introduction

Coronary artery stenting has become the most important nonsurgical coronary revascularization procedure^1^. Coronary angiography using computed tomography (CT) is limited in regards to a correct assessment of in-stent restenosis and its impact on coronary circulation, one of the major complications following the stenting procedure. Indeed, blooming artifacts impair a correct visualization of the vascular lumen. Blooming artifacts result in an apparent increase in the size of strongly attenuating objects, such as the stent’s metallic struts and vascular calcifications, leading to an apparent reduction in size of the vascular lumen. Metal strut induced blooming artifacts are more significant in stents of diameter 3 mm or less, reducing intra-stent lumen interpretability to nearly 51% compared to 81% for larger stents^2^. Furthermore, severe calcifications also induce blooming artifacts and their presence at a stent location may impair the detection of the stent itself.

The recent development of a clinically based small field-of-view (FOV) spectral photon counting CT (SPCCT) prototype achieves higher spatial resolutions than standard CT at similar energies, owing to an important reduction in detector size^3^. The higher spatial resolution is possible owing to the direct conversion photon counting detectors, which convert single x-rays to electrical pulses. Ultra-high resolution mode resulting in a 0.25 × 0.25 mm pixel size at iso-center was demonstrated recently for a photon-counting CT system^4^. This increase in spatial resolution is expected to strongly reduce blooming artifacts, potentially improving restenosis detection and interpretation^5^. Reduction in blooming artifacts should allow morphological assessment of the mesh of the stent which is expected to improve detection of stent deployment problems and stent integrity degradation^6^. Furthermore, owing to the spectral separation capabilities of photon counting, specific K-edge images can be obtained^7–12^. When a stent contains a material compatible with k-edge imaging, such as platinum, the element specific K-edge images may allow visualization of only the metal stent, eliminating all other sources of attenuation.

Thus, the objective of the study was to assess the capability of the SPCCT scanner to improve vascular lumen delineation and visualization of stent architecture, *in vitro* and in a single live rabbit, in comparison with dual-energy and standard CT.

## Results

**Figure 1:**
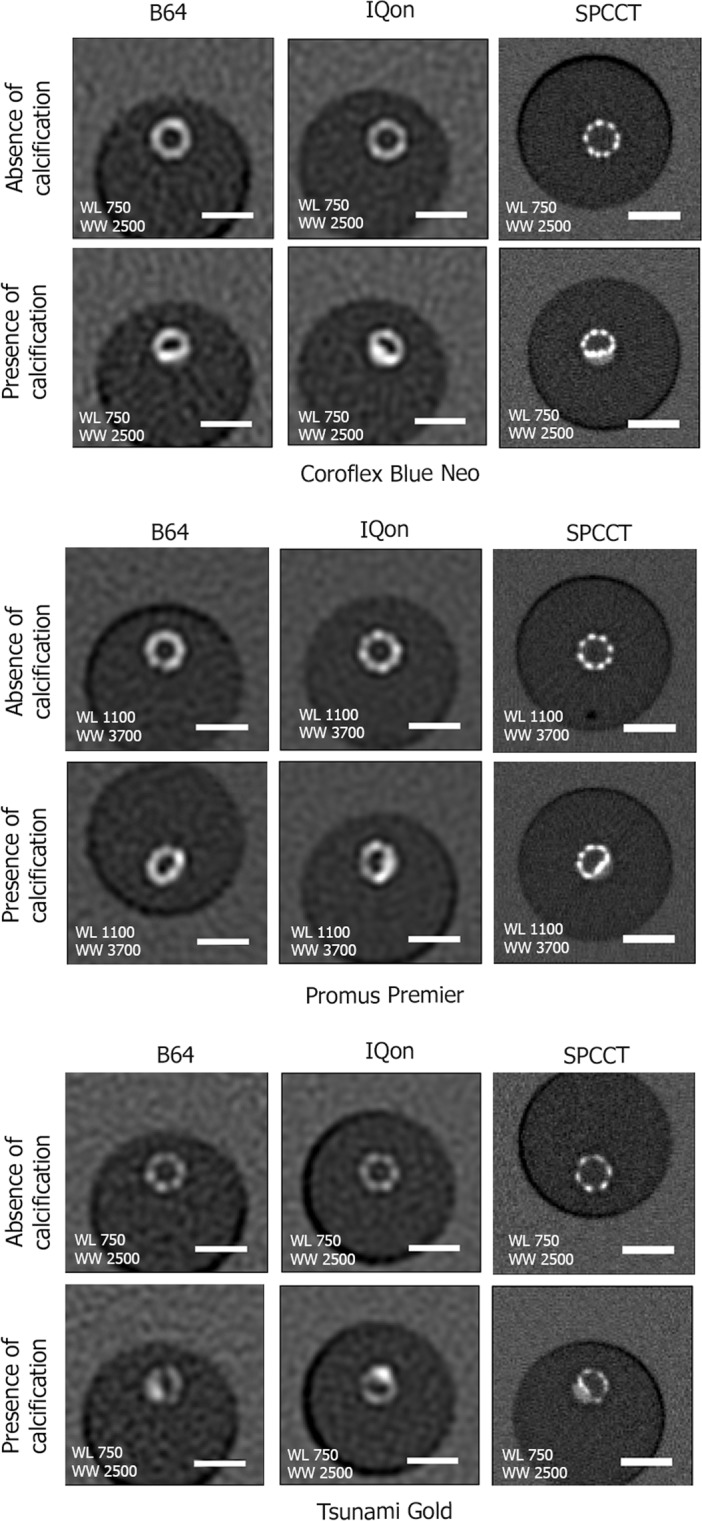
Representative conventional HU images acquired on the B64, the IQon, and the SPCCT of the three stents *in vitro* in water. Transverse views of the stents at similar locations in the absence (first row) and presence of calcification (second row). The better spatial resolution of the SPPCT system results in an improved visualization of the stent metallic struts. On the SPCCT images the stents can be visually separated from the calcification, while this separation is not possible on the B64 and IQon images due to larger detector size. A 5 mm scale bar is shown on the transverse views.

**Figure 2:**
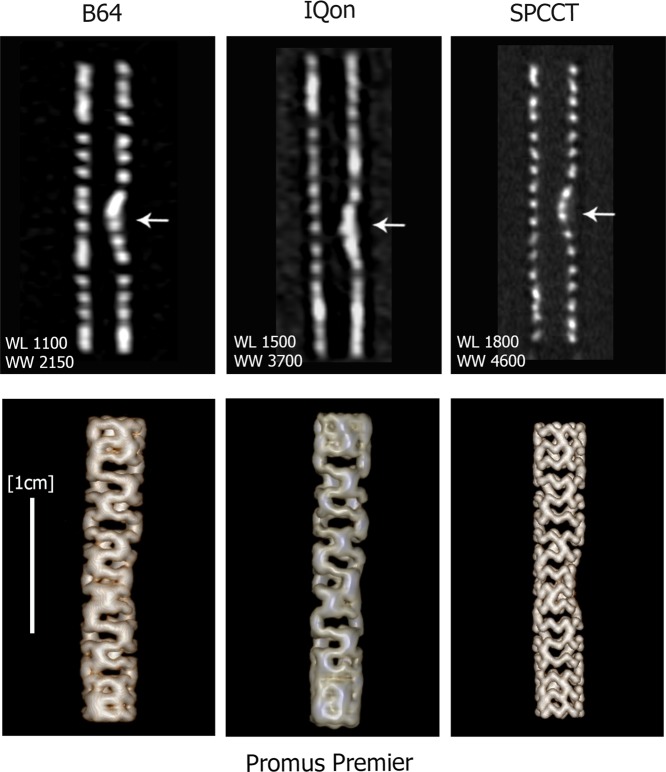
Longitudinal views of the entire length of the platinum coated stent (Promus Premier) are presented in the top row and 3D Volume Rendering are presented in the bottom row. The improved quality of SPCCT images allows clear visualization of the metallic mesh of the stent and its deformation due to the presence of the calcification insert (arrows indicate the calcification), while this is not possible for IQon and B64.

Representative conventional HU images of the stent containing phantom in water acquired on the B64, IQon, and SPCCT are presented in Fig. 1. The higher spatial resolution of SPCCT resulted in a better visualization of the intra-stent lumen. Furthermore, the visual separation of the calcification from the stent was only possible on images acquired with the SPCCT (Fig. 2, arrows indicate the calcification). 3D Volume Rendering views of the platinum coated stent (Promus Premier) performed on conventional HU images allowed clear visualization of the metallic mesh on SPCCT images (Fig. 2). Furthermore, the deformation of the metallic mesh due to the presence of the calcification was also clearly visible on the SPCCT images. However, visualization of the metallic mesh was not possible on B64 and IQon images.

**Figure 3:**
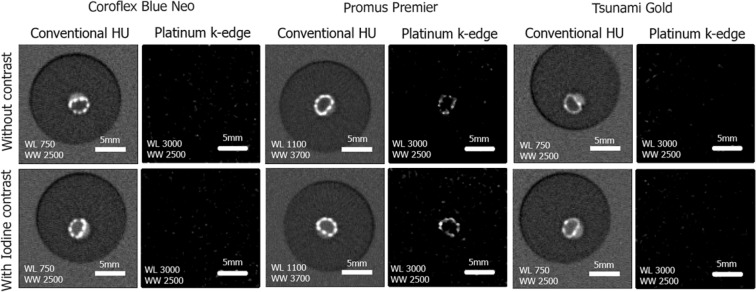
Representative conventional HU images and Pt-specific K-edge images acquired on the SPCCT in the calcification containing regions of the three different stents. The platinum coated Promus Premier stent is clearly visible on the Pt specific K-edge images while all other attenuation sources such as the calcification and the intra-luminal iodinated contrast agent are absent from these images. The clear visualization of the deformation of the metallic mesh due to the calcification is to be noted. A 5 mm scale bar is shown on each image.

The Pt-specific K-edge images allowed visualization of only the platinum coated stent (Fig. 3, Promus Premier). Signal from all other sources of attenuation such as the calcification or the luminal contrast agent were absent (Fig. 3). The other two types of stent were not visible on the Pt-specific K-edge images, except for the gold markers of Stent 3 (Stainless steel), because these two materials have similar k-edge energies (78.4 for Pt and 80.7 for Au). Furthermore, the deformed shape of the platinum-coated stent due to the presence of the calcification was clearly visible (Fig. 3 center columns).

The apparent width of the metallic struts, measured in the calcification free region, was smaller on SPCCT than both on IQon, and on B64 (Table 1). There was no difference in the apparent width of the Pt-containing stent between the conventional HU and the Pt-specific images. Similarly, the intra-stent lumen, measured in the same region, was larger on SPCCT than on IQon, and on B64. However the true size of 3.4 mm remained strongly underestimated (Table 1).

**Table 1:** *In vitro* measured apparent strut width and intra-stent lumen diameter from SPCCT, IQon, and B64.

		Coroflex Blue Neo (Co-Cr)	Promus Premier (Pt-Cr)	Tsunami Gold (Stainless Steel)
SPCCT apparent strut width (mm)	Conventional HU	0.69 ± 0.002	0.71 ± 0.002	0.69 ± 0.004
	Pt K-edge images	—	0.71 ± 0.007	—
IQon apparent strut width (mm)	Conventional HU	0.98 ± 0.06	0.99 ± 0.05	1.01 ± 0.16
B64 apparent strut width (mm)	Conventional HU	1.03 ± 0.004	1.00 ± 0.003	1.04 ± 0.009
SPCCT intra-stent lumen (mm)	Conventional HU	2.44 ± 0.01	2.28 ± 0.01	2.71 ± 0.04
IQon intra-stent lumen (mm)	Conventional HU	2.04 ± 0.08	2.21 ± 0.06	1.94 ± 0.21
B64 intra-stent lumen (mm)	Conventional HU	1.96 ± 0.006	1.9 ± 0.003	2.18 ± 0.04

**Figure 4:**
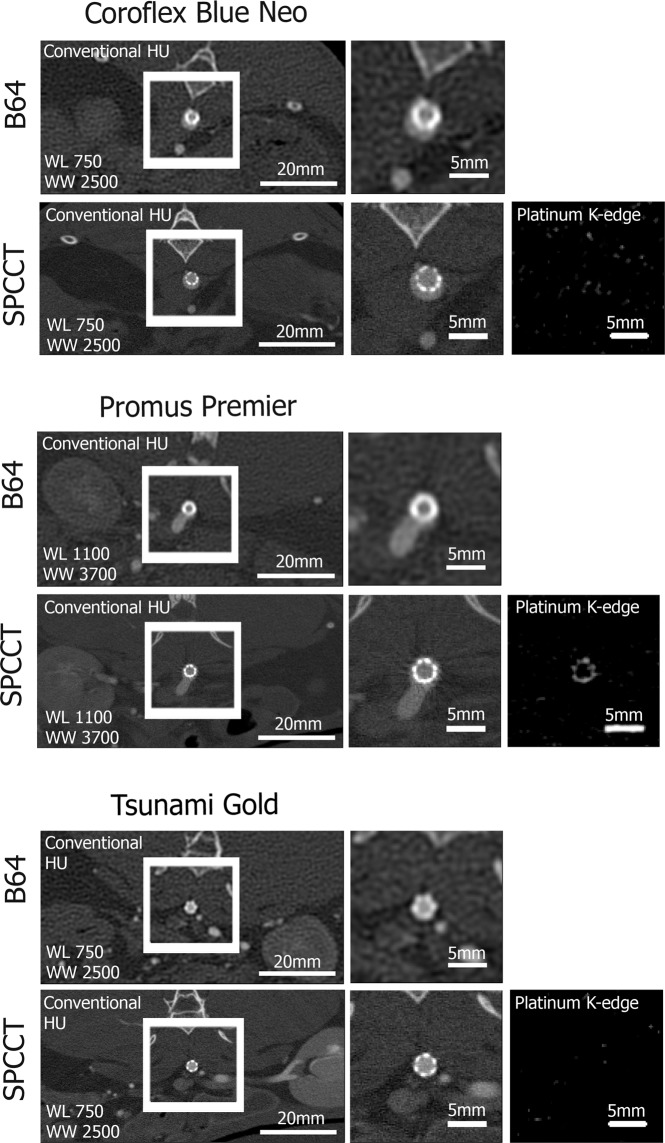
Representative *in vivo* images of the three coronary stents placed in the abdominal aorta of a NZW rabbit. Conventional HU images are presented on the two left columns of the figure. Importantly, the Pt-specific K-edge images of SPCCT (right column) enable visualization of the Pt coated stent while all other sources of attenuation are removed.

Similarly, *in vivo* images demonstrated improved visualization of the metallic mesh of stents in the abdominal aorta of the NZW rabbit (Fig. 4). Pt-specific K-edge imaging enabled exclusive visualization of the stent containing Pt only, and removal of other backgrounds and contrast media. Finally, the apparent width of metallic struts was larger *in vivo* (Table 2) compared to *in vitro* both on SPCCT and on B64.

**Table 2:** *In vivo* measured apparent strut width and intra-stent lumen diameter from SPCCT and B64.

		Coroflex Blue Neo (Co-Cr)	Promus Premier (Pt-Cr)	Tsunami Gold (Stainless Steel)
SPCCT apparent strut width (mm)	Conventional HU	0.79 ± 0.006	0.74 ± 0.003	0.75 ± 0.004
	Pt K-edge images	—	0.70 ± 0.012	—
B64 apparent strut width (mm)	Conventional HU	1.13 ± 0.009	1.11 ± 0.005	1.06 ± 0.008
SPCCT intra-stent lumen (mm)	Conventional HU	2.06 ± 0.006	1.85 ± 0.004	1.81 ± 0.003
B64 intra-stent lumen (mm)	Conventional HU	1.73 ± 0.008	1.60 ± 0.004	1.50 ± 0.008

## Discussion

In this study, we present the added value of increased spatial resolution and element specific detection of SPCCT for stented vessel imaging. Firstly, the increased spatial resolution of the SPCCT system compared to dual-energy and standard CT resulted in a significant reduction of stent related blooming artifacts that improved visualization of the intra-stent lumen. Furthermore, the increased spatial resolution allowed the evaluation of the morphology of the stent’s metallic mesh. Secondly, the capability of SPCCT to provide element specific K-edge images allowed visualization of the platinum containing stent and of the gold marker of the stainless steel stent, while all other sources of attenuation were removed from the image. These results demonstrate a high potential of SPCCT in detecting correct stent deployment.

Spatial resolution is a limiting factor for imaging small and strong attenuating objects, such as coronary stents. Neither system was capable to visualize the true size of the metallic struts (~80 microns). However, the measured width of the struts, which was measured approximately 30% more accurately on the SPCCT system than on the other systems, reflects the improved spatial resolution of the SPCCT^7,13,14^. These results are in agreement with the very recent study of Mannil and colleagues, who found a 28.8% increase in intra stent luminal diameter when comparing a photon counting detector (PCD) to a conventional energy integrating detector (EID)^13^. To be noted that in our study, different slice thicknesses were used between the SPCCT and the IQon and B64 system. While, slice thickness has an important role in blooming along the Z-axis as seen in the coronal/sagittal images, it has very little impact on the in-plane blooming. The stents were placed along the Z-axis and the apparent strut width was measured in-plane. Consequently, the slice thickness is not expected to have an influence on our finding. Also note that our acquisitions on the two clinical systems (IQon and B64) were performed using Standard Resolution mode with large focal spot (1.1 × 1.2 mm^2^) while High Resolution with small focal spot (0.6 × 0.7 mm^2^) was used on SPCCT. The justification for this is that the resolution on the B64 and IQon systems is limited by the Nyquist limit due to the detector pitch. Therefore, Standard Resolution is used in cardiac mode where scan is limited to the equivalent of 180 degrees’ parallel beam recon (high resolution requires higher sampling rate achieved by 360 degrees’ recon utilizing quarter detector offset). Futhermore, High Resolution with small focal spot is usually not used for cardiac CT in routine practice because it doesn’t provide enough X-ray flux for this fast rotation acquisition. In the case of SPCCT, the detectors are much smaller and the limiting factor becomes the focal spot size. Using large focal spot on SPCCT does not allow benefiting from the improved spatial resolution brought by the small pixel size of the photon counting detector, while small focal spot does. As SPCCT is expected to be more dose efficient than energy integrating detector CT, owing to the counting mode versus the integrating mode and smaller detectors with no electronic noise, small focal spot is expected to provide enough X-ray flux for cardiac applications^3,15^. Our *in vitro* results for B64 are in agreement with Beohar and colleagues who found that both 16- and 64-MDCT scanners underestimated lumen size by as much as 1.2 mm in stents 3 mm in diameter and strut thicknesses comparable to the ones used in the present study^16^.

Using a third generation scanner in combination with novel iterative reconstruction, visibility of on average 80% of the in-stent lumen was obtained for commonly used coronary stents inflated in plastic tubes to a diameter of 3 mm^17^. These results are very encouraging with respect to our findings as they indicate the strength of iterative reconstruction, currently not available on our SPCCT system, which is expected to further improve stent visualization. This explains the current effort to develop advanced iterative reconstruction methods such as one-step algorithms^18^. We found an average underestimation of ~1.4 mm for B64 and 0.9 mm for SPCCT. The added value of the higher spatial resolution of SPCCT is a reduction of blooming artifacts to under a millimeter. The reduction in blooming artifacts allowed morphological assessment of the mesh of the stent even in the presence of calcification. This is of particular importance in diagnosing in-stent restenosis, one of the main complications of coronary artery stenting. In-stent restenosis is due to neo-intimal hyperplasia, proliferation of smooth muscle cells along the inner vessel wall. Strong blooming artifacts impair a correct diagnosis of neointimal hyperplasia. The apparent decrease in the inner lumen diameter may be wrongly interpreted as neointimal hyperplasia. Thus the strong reduction in stent related blooming artifacts provided by SPCCT is expected to improve detection of in-stent restenosis, currently impaired by strong blooming.

*In vivo* measured apparent strut widths were larger than *in vitro*, probably due to aortic pulsation-related motion artifacts. Nonetheless, the strut width was smaller with SPCCT compared to B64 also in the live animal. This capability is expected to improve detection of stent deployment problems, such as stent malapposition, and stent integrity degradation, e.g. fractures and “jailed struts” occurring after kissing balloon inflation procedure in bifurcation regions^6^. Stent fracture occurs in almost 30% of cases, as revealed by autopsy reports^19^, contrary to the 1–2% of cases revealed by imaging due to the current limited spatial resolution of standard CT.

Importantly, the additional capability of the PCD system presented in our study is k-edge imaging. K-edge images represent specific element maps, such for example the Pt specific K-edge images of the Pt containing stent (Promus Premier) presented here. We did not obtain a statistically significant difference between the apparent strut thicknesses in the conventional HU image and the Pt K-edge image. However, this is to be expected since Platinum is a coating material. The added value of the K-edge is the specificity of the image, i.e. the only thing visible is the Platinum, while other highly attenuating objects such as the vascular calcifications are not present, and by such do not hamper the visualization of the stent. It should be noted that Au markers of the stainless steel stent were also visible in the Pt-specific K-edge images. This is due to the fact that these metals have very close atomic numbers, Z_Pt_ = 78 and Z_Au_ = 79 and thus their respective k-edge energies are very similar 78.4 keV (Pt) and 80.7 keV (Au). The high specificity of SPCCT for these materials enforces the added value of the SPCCT for coronary stent imaging. Material separation capabilities in elements such as gold was demonstrated previously for another photon-counting system using 4 energy levels^20^. It is to be noted that this advantage is only applicable to metals of relatively high atomic weights^21–25^. Stainless steel (Fe) and Co-Cr stents cannot be detected by specific K-edge imaging because their K-edge energies are smaller than 10 keV, energies that are filtered from the incident photon spectrum in CT. However, this is not a limitation, since relatively high atomic weight metals, such as Pt, have been added to stent substrates to increase their radio-opacity and thus their visibility on radiological images, and to improve their bio-compatibility.

Several drawbacks could be identified regarding the current capabilities of the SPCCT prototype with respect to cardiac imaging: no ECG-gating is available, the rotation time of 1 second is potentially long for dynamic acquisitions, and the z-collimation of 2.5 mm limits the current use to small animals. It should be noted however, that these limitations will be overcome in the next generation of the system.

## Methods

### Spectral photon-counting computed tomography (SPCCT)

The SPCCT prototype system (Philips, Haifa, Israel) is based on a standard clinical CT gantry and X-ray tube but with a direct conversion semiconductor detector technology operated in single photon-counting mode with energy discrimination^26^. The detector characteristics are presented in Table 3. The scanner has the same geometry as a standard Philips iCT system, with source to detector distance of 1040 mm and source to isocenter distance of 570 mm (magnification of 1.825). The pixel size is 0.274 × 0.274 mm^2^ at isocenter and z-coverage of ~2.5 mm (9 pixels). On this pre-clinical prototype, the maximum FOV is 168 mm and the rotation time is 1 s, enabling acquisition of 2400 projections/rotation. As references for comparison, we used two commercially available systems: a conventional Brilliance 64 CT (B64, Philips, Cleveland, USA) and a dual-energy (DE) CT (IQon, Philips Healthcare, Haifa, Israel). The IQon uses dual-layer detector to segregate low-energy photons from high-energy photons resulting in two raw-data sets that are used in reconstruction algorithms. This concept is different from other DECT systems in that it allows the CT user to process spectral data without need to select a specific dual-energy protocol prior to an examination.

**Table 3:** Detector characteristics.

Pixel Size	500 µm pitch, flip-chip to sensor
Observable count rate	>10 Mcps/pixel, Paralyzable
Input referred noise	<350 ${e}^{-}$
Leakage compensation	Static 200 nA Dynamic 60/600 nA 3 dB at 10 kHz
Thresholds	5
Energy range	>160 keV
Energy resolution	0.5 keV/LSB
Frame rate	>10 kHz, zero dead-time

### Imaging protocols

Three types of coronary stents of different metal composition: Cobalt-Chromium (Stent1), Platinum (Pt)-Chromium (Stent2), and Stainless steel with gold (Au) markers (Stent3) (see Table 4) were imaged using the SPCCT prototype, the IQon and the B64 systems *in vitro*, and the SPCCT and the B64 *in vivo*, all with 120 kVp and 100 mAs tube settings. IQon and SPCCT have same tube filtration while B64 has additional Ti filter resulting with 25–30% lower dose.

**Table 4:** Description of the three types of coronary stents used in the study.

	Stent 1	Stent 2	Stent 3
Commercial Name	Coroflex Blue Neo (Braun, Melsungen AG, Germany)	Promus Premier Monorail (Boston Scientific, France)	Tsunami Gold (Terumo Europe NV)
Composition	Cobalt-Chromium	Platinum (Pt)-Chromium	Stainless steel with gold markers
Strut thickness (mm)	0.081	0.060	0.080
Length (mm)	19	24	10

On the B64 and IQon, the acquisitions were performed in spiral mode using 64 × 0.625 mm collimation, gantry rotation time of 1 second, pitch of 0.56, and a large focal spot on the X-ray tube (standard resolution mode). On the SPCCT, the acquisitions were performed in axial step and shoot mode with 9 × 0.274 mm collimation, rotation time of 1 second and with small focal spot on the X-ray tube (high Resolution mode) to benefit from the potential spatial resolution improvement allowed by the small detector size.

The reconstructed FOV and voxel size were: 160 mm and 0.2 × 0.2 in plane pixel size and slice thickness of 0.25 mm on SPCCT using a “detailed” filter; and 154 mm and 0.2 × 0.2 in plane pixel size, effective slice thickness of 0.67 mm and increment of 0.33 mm on IQon and B64, using the YA sharp recon kernel. For the SPCCT the photon counting thresholds were adjusted to allow for simultaneous detection of iodinated contrast material and platinum K-edge.

Reconstructions of SPCCT acquired data were performed using maximum-likelihood processing method of counting data to compute several basis material sinograms (water, Iodine, and Platinum)^27^. The individual sinograms are then reconstructed to obtain conventional HU images, water and Iodine material density maps and Platinum specific K-edge images.

#### *In vitro* imaging

The stents were deployed inside individual plastic tubes (internal lumen diameter of 3.5 mm). Presence of calcification was simulated by using a mixture of Calcium Phosphate powder (25%), flour (45%), and water (30%). Small spheres (1–2 mm in diameter) were obtained from the mixture and allowed to dry for 24 hours and were then placed inside the plastic tubes before stent deployment. To assess the impact of the presence of vascular contrast agents on stent imaging, tubes were subsequently filled with water and with a standard iodinated contrast agent (a concentration of ~11 mg/ml of elemental iodine (I)). The entire length of the stents was imaged for each type of filling on the three systems.

#### *In vivo* imaging

This study was approved by the local Institutional Animal Care and Use Committee (Council Directive No. 2010/63/UE on the protection of animals used for a scientific purpose) under the authorization n° APAFIS#1732-2015091411181645v3 and performed under relevant guidelines and regulations.

Similar stents were sequentially deployed in the abdominal aorta of one New Zealand White rabbit (NZW, male, 3.2 kg) while under full anesthesia achieved with an intramuscular injection of medetomidine (0.4 mg/kg) and ketamine (20 mg/kg). An incision was performed at the level of the left hind paw to reach the iliac artery. The guidewire supporting the stent was then inserted in the artery up to the abdominal aorta at the level of the diaphragm and the first stent was deployed, followed by the other two stents. Stent placement was verified by DSA. During the procedure, the animal’s heart rate and oxygen saturation was monitored. At the end of the procedure the incision was sutured and anti-inflammatory cream was applied locally and the animal was allowed to recover for 2 weeks before the first imaging session.

The animal underwent two imaging sessions, one on SPCCT and one on B64. On SPCCT, cross-sectional views of each stent were acquired before and after manual intravenous injection of a standard iodinated contrast agent (400 mg/ml of elemental I, IOMERON, Bracco, Italy). The three stents were imaged individually, each one approximately 5 seconds after the administration of 5 ml of IOMERON, equivalent to a high clinical dose of 1.6 ml/kg, with at least a 5 minute interval between the injections, to allow the blood concentration of iodine to decrease. On B64, one injection of 5 ml was performed and the entire length of the abdominal aorta was imaged approximately 5 seconds after the manual injection.

### Image analysis

To evaluate the stent-related blooming artifacts, automatic measurements of the apparent width of the metallic struts and of the intra-stent lumen diameter were performed on images acquired *in vitro* in the calcification free region and *in vivo*, using in house developed code as follows: the center of the stent cross-section was identified on 8 consecutive images and intensity profiles were drawn radially center-out using 5° increments, resulting in 576 intensity profiles. The apparent strut width was obtained as the full-width at half maximum (FWHM) value of a Gaussian curve fitted to the interpolated intensity profile using least square fitting. Profiles located at a 180° angle from each other were paired and used to compute the inter-stent lumen diameter following identification of points corresponding to half the maximum intensity on each side of the curve. Then, the inter-stent lumen diameter was defined as the distance between the closest identified points with respect to the center (288 measurements). This analysis was performed on conventional HU images and on Pt-specific K-edge images acquired in water *in vitro* and before contrast agent administration *in vivo*.

Data is presented as mean ± standard error of the mean.

## Conclusion

SPCCT enables improved lumen delineation and visualization of stent metallic mesh owing to increased spectral resolution and increased spatial resolution that resulted in a significant reduction of blooming artifacts compared to dual-energy and conventional CT. Platinum specific K-edge images allow validation of correct deployment of Pt containing stents.
